# Optimizing early child development for young children with non-anemic iron deficiency in the primary care practice setting (OptEC): study protocol for a randomized controlled trial

**DOI:** 10.1186/s13063-015-0635-z

**Published:** 2015-04-02

**Authors:** Kawsari Abdullah, Kevin E Thorpe, Eva Mamak, Jonathon L Maguire, Catherine S Birken, Darcy Fehlings, Anthony J Hanley, Colin Macarthur, Stanley H Zlotkin, Patricia C Parkin

**Affiliations:** Pediatric Outcomes Research Team, Division of Pediatric Medicine, Department of Pediatrics, The Hospital for Sick Children, 555 University Avenue, M5G 1X8 Toronto, Canada; Institute of Health Policy, Management and Evaluation, University of Toronto, 155 College Street Suite 425, M5T 3 M6 Toronto, Canada; Li Ka Shing Knowledge Institute of St Michael’s Hospital, 30 Bond Street, M5B 1 W8 Toronto, Canada; Dalla Lana School of Public Health, University of Toronto, 155 College Street 6th floor, M5T 3 M7 Toronto, Canada; Department of Psychology, The Hospital for Sick Children, 555 University Avenue, M5G 1X8 Toronto, Canada; Department of Pediatrics, St Michael’s Hospital, University of Toronto, 30 Bond Street, M5B 1 W8 Toronto, Canada; Child Health Evaluative Sciences, The Hospital for Sick Children Research Institute, Peter Gilgan Centre for Research and Learning (PGCRL), 686 Bay Street, M5G 0A4 Toronto, Canada; Division of Developmental Pediatrics, Holland Bloorview Kids Rehabilitation Hospital, and Bloorview Research Institute, 150 Kilgour Road, M4G 1R8 Toronto, Canada; Department of Pediatrics, Faculty of Medicine, University of Toronto, 1 King’s College Circle, Medical Sciences Building, Room 2109, M5S 1A8 Toronto, Canada; Department of Nutritional Sciences, University of Toronto, FitzGerald Building, 150 College Street, Room 316, M5S 3E2 Toronto, Canada; Division of Endocrinology, Department of Medicine, Faculty of Medicine, University of Toronto, 1 King’s College Circle, Medical Sciences Building, Room 2109, M5S 1A8 Toronto, Canada; Research Institute, The Hospital for Sick Children, Peter Gilgan Centre for Research and Learning (PGCRL), 686 Bay Street, M5G 0A4 Toronto, Canada; Division of Gastroenterology, Hepatology and Nutrition, Department of Pediatrics, The Hospital for Sick Children and University of Toronto, 555 University Avenue, M5G 1X8 Toronto, Canada

**Keywords:** NAID, IDA, Screening, MSEL, ELC, Serum ferritin, Hemoglobin, Pre-school children

## Abstract

**Background:**

Three decades of research suggests that prevention of iron deficiency anemia (IDA) in the primary care setting may be an unrealized and unique opportunity to prevent poor developmental outcomes in children. A longitudinal study of infants with IDA showed that the developmental disadvantage persists long term despite iron therapy. Early stages of iron deficiency, termed non-anemic iron deficiency (NAID), provide an opportunity for early detection and treatment before progression to IDA. There is little research regarding NAID, which may be associated with delayed development in young children. The aim of this study is to compare the effectiveness of four months of oral iron treatment plus dietary advice, with placebo plus dietary advice, in improving developmental outcomes in children with NAID and to conduct an internal pilot study.

**Methods/Design:**

From a screening cohort, those identified with NAID (hemoglobin ≥110 g/L and serum ferritin <14 μg/L) are invited to participate in a pragmatic, multi-site, placebo controlled, blinded, parallel group, superiority randomized trial. Participating physicians are part of a primary healthcare research network called TARGet Kids! Children between 12 and 40 months of age and identified with NAID are randomized to receive four months of oral iron treatment at 6 mg/kg/day plus dietary advice, or placebo plus dietary advice (75 per group). The primary outcome, child developmental score, is assessed using the Mullen Scales of Early Learning at baseline and at four months after randomization. Secondary outcomes include an age appropriate behavior measure (Children’s Behavior Questionnaire) and two laboratory measures (hemoglobin and serum ferritin levels). Change in developmental and laboratory measures from baseline to the end of the four-month follow-up period will be analyzed using linear regression (analysis of covariance method).

**Discussion:**

This trial will provide evidence regarding the association between child development and NAID, and the effectiveness of oral iron to improve developmental outcomes in children with NAID. The sample size of the trial will be recalculated using estimates taken from an internal pilot study.

**Trial registration:**

This trial was registered with Clinicaltrials.gov (identifier: NCT01481766) on 22 November 2011.

**Electronic supplementary material:**

The online version of this article (doi:10.1186/s13063-015-0635-z) contains supplementary material, which is available to authorized users.

## Background

### Prevalence of iron deficiency and iron deficiency anemia

Nationally representative data from the United States indicates a prevalence in children one to three years of age of 9% and 3%, for iron deficiency and iron deficiency anemia (IDA), respectively [1,2]. European children (one to three years of age) have been reported to have similar rates: 5 to 20% for iron deficiency and 3 to 9% for IDA [3]. Although there are no similar nationally representative data for Canadian children, regional studies suggest a similar rate that has led *Hartfield* to conclude that ‘iron deficiency is an inadequately addressed and significant public health problem among Canadian infants and children’ [1,2,4-13].

### Current recommendations regarding screening for iron deficiency anemia in primary care practice

The American Academy of Pediatrics (AAP) has concluded that universal screening for anemia should be performed, with determination of hemoglobin concentration, at approximately one year of age [14]. Universal screening should also include an assessment of risk factors associated with iron deficiency: prematurity, low birth weight, exposure to lead, exclusive breastfeeding beyond four months of age without supplemental iron, weaning to whole milk or complementary foods that do not include iron-fortified cereals or foods naturally rich in iron, feeding problems, poor growth, and low socioeconomic status. Selective screening should be performed at any age when these risk factors for iron deficiency and IDA have been identified [13]. However, both the Canadian Task Force on the Periodic Health Examination (1994) and the United States Preventive Services Task Force (2006) have concluded that the evidence is insufficient to recommend for or against routine screening for IDA in asymptomatic children [15,16]. These guidelines have focused on IDA, but have not addressed screening for non-anemic iron deficiency (NAID). Furthermore, experts have highlighted the lack of evidence and paucity of high quality investigations on which to base guidelines relevant to iron deficiency [17].

### Current evidence on early child development and iron deficiency anemia

Studies of the outcomes of IDA have been largely conducted in developing countries due to its high prevalence. Poor developmental outcomes of IDA, which persist long term despite iron therapy, have been summarized in two recent reviews of longitudinal observational studies [14,18]. A Cochrane systematic review that was updated in 2013 examined the effectiveness of longer term iron treatment, and demonstrated improvements in developmental outcomes for the IDA group receiving oral iron treatment, compared to those receiving placebo [19,20]. Studies included in the review also demonstrated that it is possible to detect meaningful changes in tests of children’s cognition over a four-month-period. Although the authors of the Cochrane review concluded that there is an urgent need for further randomized trials in children with IDA with long term follow-up periods, there have been no recent trials; most investigators currently consider the question of the association between IDA and development to lack equipoise.

### Current evidence on early child development and non-anemic iron deficiency

The authors of the current protocol (KA and PCP) have recently undertaken a systematic review of the literature regarding the effectiveness of oral iron treatment to improve the developmental and hematologic outcomes of young children with NAID [21]. From the titles of 743 articles, a full-text review was completed on 46, and two randomized controlled trials were found for preschool-aged children with NAID treated with oral iron, 3 to 6 mg/elemental iron/kg daily, versus no treatment [22,23]. For both studies, the primary objective was to study children with IDA; however, both included children with NAID randomized to oral iron or no treatment, and this data was available. The first study (n = 29, conducted in Indonesia, published in 1993) showed no statistically significant difference between groups in the post-treatment developmental score [23]. The second study (n = 40, conducted in Turkey, published in 2004) showed a statistically significant difference between groups in the post-treatment mental developmental score, but not in the psychomotor developmental score [22]. Meta-analysis was not possible due to significant heterogeneity. Both studies showed moderate risk of bias due to insufficient information regarding allocation concealment and inadequate reporting of adjustment for covariates (notably, sociodemographic variables); furthermore, mothers were not blinded in the second trial as a placebo was not used. We have concluded that the effectiveness of oral iron treatment in children with NAID to improve developmental outcomes remains in question, and with this equipoise it is ethical and urgent to conduct such a trial. The results of a high quality, adequately powered trial, conducted in a developed country, will begin to establish an evidence base for screening for iron deficiency, with an aim to improve developmental outcomes.

### Establishment of the TARGet kids! research network

The investigators of this protocol (PCP, CSB, and JLM) have established a program of research aimed at advancing the scientific basis for preventative primary healthcare for young children. This includes development of a primary care practice-based research network called TARGet Kids! to conduct observational and interventional studies in early childhood to improve child health outcomes through primary prevention [24]. It represents an innovative collaboration between child health researchers in the Faculty of Medicine at the University of Toronto, and children’s primary care physicians (pediatricians and family physicians) from the Department of Pediatrics and the Department of Family and Community Medicine, also at the University of Toronto.

Between June 2008 and September 2013, more than 5,000 children, under six years of age have been enrolled in the TARGet Kids! Cohort, with a collection of non-invasive measures, including questionnaires, and physical measures, and laboratory testing has been completed in more than 2,500 children [24]. We have leveraged existing TARGet Kids! infrastructure, collaborations, research personnel, and data management system to carry out this randomized controlled trial.

### Developing a focus in child development

Since September 2008, TARGet Kids! data collection includes the measurement of child behavior (temperament) through a parent-completed questionnaire (Children’s Behavior Questionnaire). We have found that children aged between three and five years, with high ‘negative affect’, are at higher nutrition risk [25]. In the current trial, we aim to expand our focus in child development. In order to reach this goal we have established collaborations with researchers in child development. Including a focus in child development in the overall TARGet Kids! initiative is a strategic opportunity, given the importance of healthy developmental trajectories as a critical outcome measure for children [26,27].

### Rationale for a focus of research to optimize early child development through screening for non-anemic iron deficiency in the primary care practice setting

Iron status can be considered as a continuum from normal iron status, to iron deficiency without anemia, and finally, IDA [28]. NAID is the early latent stage of iron deficiency. In NAID, the amount of stored iron is reduced, but the amount of functional iron may not be affected; thereby children who have NAID have no iron stores to mobilize if the body requires more iron [28]. If iron is not provided, NAID may progress to IDA, the most severe form of iron deficiency. This natural history provides an opportunity for early detection through screening by physicians in primary care settings.

Early detection of NAID presents an important opportunity to provide effective interventions which may improve child developmental outcomes. There are critical gaps in knowledge regarding the effectiveness of iron interventions in improving the development of young children with NAID. This proposal can address this gap, and also assess the feasibility of screening for NAID in the primary care setting.

### Trial objectives and hypotheses

The primary objective of this trial is to assess the effectiveness of four months of oral iron plus dietary advice versus placebo plus dietary advice, in children with NAID aged 12 to 40 months, to improve their developmental outcomes. We hypothesize that children receiving four months of oral iron plus dietary advice will have better developmental outcomes than those who receive placebo plus dietary advice.

Secondary objectives include comparing four months of oral iron treatment plus dietary advice versus placebo plus dietary advice, for the following secondary outcomes: laboratory measures of iron indicators (serum ferritin and hemoglobin), and behavioral outcomes such as temperament, in children with NAID.

## Methods/Design

### Study design

The ‘Optimizing Early Child Development for Young Children with Non-Anemic Iron Deficiency in the Primary Care Practice Setting’ (OptEC) study is designed as a multi-site, pragmatic, placebo controlled, superiority randomized trial. From a screened cohort, children identified with NAID are randomly allocated in a 1:1 ratio to each treatment group. This trial has been designed along the pragmatic end of the pragmatic-explanatory continuum, since it was designed to primarily inform decision making [29]. Specifically, eligibility criteria, participant compliance, intensity of follow-up, and primary analysis follow pragmatic approaches, while practitioner expertise and adherence, intervention, and follow-up of outcomes follow approaches midway along the pragmatic-explanatory continuum. This protocol was designed following the Standard Protocol Items: Recommendations for Interventional Trials (SPIRIT) guidelines (see Additional file 1), and results will be reported according to the 2008 Consolidated Standards for Reporting Trials (CONSORT) guidelines for pragmatic trials [30,31].

### Setting

This multi-site study is being conducted in the offices of primary care practices participating in the TARGet Kids! practice-based research network. The Optimizing Early Child Development in the Primary Care Practice Setting: Pragmatic Randomized Trial of Iron Treatment for Young Children with Non-anemic Iron Deficiency (OptEC) trial is embedded in the TARGet Kids! cohort. The screening cohort includes all eligible children attending their well-child visit with their primary care physician. To date, TARGet Kids! practice sites are located in Toronto, Ontario. There are currently seven pediatric practices and three family medicine practices involved in patient recruitment. Each practice has between three and 10 practicing physicians.

### Participants

Children aged 12 to 40 months, whose parents consent to participate in the TARGet Kids! study, constitute the screening cohort of this trial. These parents are given a letter containing a short description of the OptEC trial. This letter states that if their child is found eligible, the parents will be contacted by phone to invite them to participate in the OptEC trial.

### Eligibility for randomization

All participants in the screening cohort undergo laboratory screening for iron deficiency, and children are assigned to one of three categories based upon the results of their hemoglobin, serum ferritin, and C-reactive protein (CRP):

1. NAID, determined by hemoglobin ≥110 g/L, serum ferritin <14 μg/L, and CRP <10 mg/L.
2. IDA, determined by hemoglobin <110 g/L, serum ferritin <14 μg/L, and CRP <10 mg/L.
3. IS, determined by hemoglobin ≥110 g/L, serum ferritin ≥14 μg/L, and CRP <10 mg/L.

**Figure 1:**
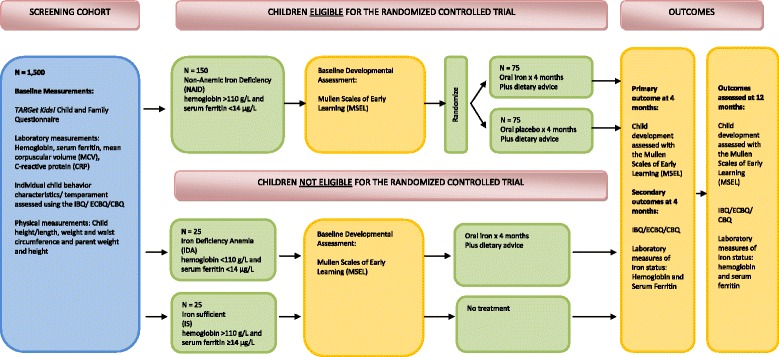
**Study schematic of the ‘Optimizing Early Child Development in the Primary Care Physician Practice Setting: Pragmatic Randomized Trial of Iron Treatment for Young Children with Non-Anemic Iron Deficiency’ (OptEC) trial.** IBQ/ECBQ/CBQ: Infant Behavior Questionnaire/Early Childhood Behavior Questionnaire/Children’s Behavior Questionnaire.

In this trial, only children diagnosed with NAID are randomized to the intervention and control groups. The other two groups (IDA and IS) are non-randomized comparators (Figure 1: schematic of study plan). However, for all three groups, we exclude children with any of the following: a CRP level ≥10 mg/L; a previously diagnosed developmental disorder; a genetic, chromosomal or syndromic condition; chronic medical condition (with the exception of asthma and allergies), including chronic anemia, iron deficiency, or recent oral iron supplementation or treatment; prematurity, with a gestational age of less than 35 weeks; low birth weight less than 2,500 g; attending the office for an acute illness, such as a viral illness, or other health concern other than for a well-child assessment; any contraindications to receiving elemental iron; the use of any natural health product containing the same medicinal ingredient(s) as the investigational product; or if English is not spoken to the child in the home or in a child care setting.

### Intervention and control

Children with NAID are randomized to receive either oral iron treatment (6 mg elemental iron/kg/day) or placebo (equivalent volume) in two divided doses for four months [32]. A drop-based formulation containing ferrous sulfate (Fer-In-Sol™, Mead Johnson Nutrition, Evansville, Indiana, USA) was chosen as the active agent to facilitate ease of administration to young children. The placebo is developed by the Hospital for Sick Children compounding pharmacy, and similar in color and taste to the active agent.

Children in both the oral iron and placebo group are also given dietary advice to improve iron intake. A guideline for improving iron intake was developed to serve this purpose (see Additional file 2: iron intake guideline). It is based on the recommendations in Canada’s Food Guide and the Hospital for Sick Children’s online guideline to improve iron intake in children [33,34]. Dietary advice includes recommendations on the varied sources of foods containing high amounts of iron, foods that increase and inhibit iron absorption, and dietary habits that may prevent iron deficiency (such as maximum daily cow’s milk intake and limiting the intake of juice).

Concomitant interventions permitted include over the counter multivitamins which do not contain iron; those prohibited include additional over the counter iron and prescription iron. To monitor adherence at the end of the trial, parents are asked to return bottles, and the amount of iron administered is calculated based on the volume of solution remaining. Parents in both groups are advised of possible adverse effects of oral iron (constipation and black stools), which are reversible and non-harmful, and are encouraged to remain compliant if these develop. A participant information sheet that contains information on the study drug is provided to parents who agree to participate in the trial. No specific criteria are being used for discontinuation or modification of the interventions, as the dose of iron is within the safe and recommended dosages for children [32].

Assignment of the interventions to the treatment groups is randomized and the randomization is stratified by clinic site. Block randomization is generated with blocks of variable sizes to ensure that group sizes are similar at the end of each block [35]. The randomization sequence for each clinic site is generated using computer-generated random numbers by KT (biostatistician). Allocation concealment is achieved by having the pharmacy department at the Hospital for Sick Children prepare the treatment and placebo in sealed, serially numbered bottles of similar appearance and weight, according to the allocation sequence. Parents, attending physicians, laboratory personnel, and study personnel conducting the outcome assessments, and data analysts and investigators are blind to the group allocation. Study medication and placebo are supplied in bottles that look identical, and the appearance, consistency, and taste of the liquid are similar. Group allocation will remain concealed until the final data analysis is performed.

If a subject in the randomized study deteriorates or has persistent, severe, bothersome side effects then unblinding may be necessary. Emergency unblinding will only be done when the clinical treatment of the patient will be different by knowing which arm of the study the patient was on. The physician caring for the subject will contact the principal investigator or co-investigator first to discuss the unblinding procedure. The study investigators should remain blinded if possible.

### Non-randomized children

Children who are identified with IDA and IS constitute the non-randomized part of the OptEC trial (Figure 1: schematic of study plan). Children with IDA receive oral iron treatment, 6 mg elemental iron/kg/day, in two divided doses for four months plus dietary advice, which is considered standard of care. Children with IS do not receive any intervention.

### Outcomes and measures

The primary outcome for the OptEC trial is the Early Learning Composite (ELC), assessed using the Mullen Scales of Early Learning (MSEL). The MSEL measures five distinct developmental skills: gross motor and four ‘cognitive’ skills (fine motor, visual reception, receptive language, and expressive language). The four cognitive skills are summarized and converted into age-adjusted normalized ELC scores, which has a mean of 100 and a standard deviation of 15 [36]. Secondary outcomes include two laboratory measures of iron status (hemoglobin and serum ferritin levels) and measurement of individual child behavior characteristics (known as child temperament), which is assessed using age-appropriate parent reported questionnaires: the Infant Behavior Questionnaire (IBQ), the Early Childhood Behavior Questionnaire (ECBQ), or the Children’s Behavior Questionnaire (CBQ).

### Rationale for selection of outcome measures for this study

#### Assessment of cognitive and motor function

The MSEL is an individually administered scale for assessment of development that may be applied to young children from birth to 68 months [36]. The standardization sample mainly represented Caucasian American children from urban communities with middle class socioeconomic status. All scales of this tool have adequate test floors: that is, a child of any age can score at least two standard deviations below their respective means. Administration of the MSEL requires approximately 30 to 40 minutes. The psychometric properties of the MSEL have been shown to be adequate [37].

The MSEL has been used to assess development in several pediatric conditions, including autism spectrum disorders, profound hearing loss, genetic conditions, biliary atresia, language delay, and congenital hypothyroidism [38-41]. Although the Bayley Scales of Infant Development (BSID) is the most common development assessment tool used in previous studies of iron deficiency, a recent study has demonstrated that the BSID may underestimate developmental delay in children [19,42]. For the current study, considering its strong psychometric properties, similarity of the population used in this trial with the standardization population, and its extensive use in the pediatric population, the MSEL has been selected as the scale to assess cognition and motor development in children.

#### Assessment of children’s social and emotional behavior

There is a reported association between iron deficiency and altered infant social-emotional behavior, including shyness, frustration, poor engagement, soothability, and affect, as measured in laboratory settings [43-45]. We have selected three age-appropriate validated parent-completed questionnaires for the assessment of individual characteristics of child behavior, specifically known as child temperament: the IBQ for infants aged 12 to 17 months old, the ECBQ for toddlers aged 18 to 36 months), and the CBQ for preschoolers aged 37 to 72 months [46,47]. Three dimensions of temperament are measured: negative affectivity, surgency or extraversion, and effortful control. Our research team has recently studied pre-school children’s temperament using the CBQ and found children who scored highly on the negative affectivity scale had significant association with higher nutrition risk, suggesting that child temperament may be associated with nutritional disorders such as obesity and iron deficiency [25].

#### Laboratory measures of iron status (hemoglobin and serum ferritin)

The hemoglobin cut-off level of >110 g/L distinguishes anemia from non-anemia in children under five years of age [1,2,18]. In adults, serum ferritin has been found to be the most appropriate test for diagnosis of IDA, with a cut-off level of ≤15 μg/L, and a recommended cut-off level of <10 or <12 μg/L for children [14,48,49]. Our laboratory measures serum ferritin using a Roche modular platform (Roche Diagnostics Limited, Rotkreuz, Switzerland) and hemoglobin is measured using the Sysmex platform (Sysmex Canada, Mississuaga, ON, Canada) [50,51]. The Roche modular platform uses a corrective method to analyze serum ferritin [52]. This correction increases the cut-off level of serum ferritin for children to <14 μg/L. Hence, for operational purposes, our trial uses this corrective value of <14 μg/L of serum ferritin to distinguish between iron deficiency and iron sufficiency. Other population-based research, such as the National Health and Nutrition Examination Survey (NHANES), uses similar serum ferritin levels to identify iron deficiency in children [52].

Because serum ferritin is an acute phase reactant, concurrent measurement of CRP has been recommended [14]. An elevated level of CRP suggests that the ferritin level may be falsely elevated [53]. Hence, we excluded these children from our sample.

### Participant timeline

Participants (NAID, IDA, and IS) are assessed at three time points: baseline, four, and 12 months after the baseline visit. At the baseline assessment children are first screened for serum ferritin, hemoglobin, and CRP to identify their iron status. A venous blood sample (3 mL) is used to measure the baseline iron status. According to the results of the screening laboratory test, those who are eligible and agree to participate in the OptEC trial are asked to come back to the physician’s office to complete their baseline developmental testing (MSEL). Other baseline data include a parent-completed, standardized data collection form based on questions used in the Canadian Community Health Survey [54]. The following data is collected: child and family characteristics (including demographic data, socioeconomic status, ethnicity, family structure, child care, and familial illnesses), child diet, physical activity, and health. Individual child behavior characteristics known as child temperament are also assessed using the IBQ, ECBQ, or CBQ. Measurement of height and length, weight, and waist circumference of participants and their accompanying parent is performed using standardized anthropometric protocols [55].

After the baseline assessment, intervention is provided for four months. The four-month follow-up visit is considered the time of the primary outcome assessment, and includes measurement of: development (MSEL), temperament (IBQ, ECBQ, or CBQ), and anthropometric and laboratory tests (serum ferritin, hemoglobin, and CRP). Parents are asked to complete a follow-up questionnaire (Additional file 3: four-month follow-up form) that collects data related to administration of the study drug, causes of non-adherence, adverse effects, and an approximate per week rate of missed doses of the study drug. The questionnaire also collects data on children’s frequency of illness during the 4 month period.

The 12-month follow-up visit is a non-intervention longitudinal follow-up visit for children in all three iron groups (Table 1: Schedule of procedures, assessments, and interventions). At the 12-month follow-up visit developmental, temperament, anthropometric, and laboratory (serum ferritin, hemoglobin, and CRP) testing is performed.

**Table 1:** **Schedule of procedures, assessments, and interventions**

		**Study period**			
	**Pre-randomization**	**Intervention**		**Post-intervention follow-up**	**Post-intervention follow-up**
**Time point**	***1 weeks***	**0**	***4 months***	***4 months***	***12 months***
**Subjects**	**Screening Cohort N = 1500**	**RCT subjects n = 150**	**RCT subjects n = 150**		
INTERVENTIONS:					
Intervention		X	X		
Control		X	X		
PROCEDURES: Informed consent	X				
Eligibility screen	X				
Randomization		X			
ASSESSMENTS:					
*TARGet Kids!* Child and Family Questionnaire	X				
Laboratory measurements: Hemoglobin, serum ferritin, mean corpuscular volume (MCV), C-reactive protein (CRP)	X			X	X
IBQ (12-17 months infants)/ECBQ (18-36 months children)/CBQ (37-72 months children)*	X			X	X
Physical measurements: height and weight, waist circumference	X			X	X
Mullen Scales of Early Learning (MSEL)		X		X	X
4 month follow-up questionnaire				X	

*IBQ: Infant Behavior Questionnaire; ECBQ: Early Childhood Behavior Questionnaire; CBQ: Children’s Behavior Questionnaire.

### Recruitment and retention

The TARGet Kids! consent form informs parents that their child, once enrolled in the cohort, may be eligible for a trial (such as the OptEC trial). Based on the laboratory results of the screening cohort, research assistants (RA) are instructed to contact the parents of potential participants by phone and request that they return to the physician’s office if they agree to participate. The RAs are provided with a scripted telephone dialogue in order to standardize the patient recruitment process. At the physician’s office, the RA reviews the consent form with the parent, and the clinic nurse reviews the iron status of the child with the parent and obtains informed written consent. The family has the opportunity to ask the RA, the pediatrician, and/or clinic nurse any questions at any time. For families who provide consent, RAs conduct follow-up phone calls to ensure that the child is taking the study drug and address any questions parents may have. Since the study drug is given for four months, after the four-month follow-up visit no further contact with the participant is conducted before the 12-month follow-up visit. Two weeks prior to the 12-month follow-up visit the RA contacts the participant via phone and schedules the visit.

### Data collection methods

Questionnaire data and physical measurements are collected by the RAs embedded in the practices. The RAs have been trained to ensure accuracy of data collection and the questionnaires have been extensively pilot tested. Blood work is obtained by trained personnel according to the arrangements established at each of the practice sites. The RA is responsible for ensuring the blood is delivered to Mount Sinai Services ((MSS) Toronto, Canada) for laboratory testing. MSS provides customized laboratory and research services to pharmaceutical and biotech companies, and researchers. Laboratory results are sent electronically to the data management center, as well as faxed to the practicing physicians offices. Children eligible for the randomized trial return to the physician’s office where developmental testing is completed using the MSEL, administered by a trained psychometrist under the supervision of a registered psychologist. At the four and 12-month follow-up visits, the RA is responsible for ensuring that the questionnaires, and physical and laboratory measures are completed. The psychometrist completes the MSEL.

### Data management

The Applied Health Research Centre (AHRC) of the Keenan Research Centre, Li Ka Shing Knowledge Institute of St Michael’s Hospital, University of Toronto, serves as the data management centre for this trial. AHRC employs state-of-the-art web-based data management software RAVE™ (version 5.6.3, Medidata Solutions Inc., New York, USA), which uses secure encrypted web-based data capture technology and is the repository for data collected during this study. It has user configurable workflows, sophisticated case report form (CRF) design, complex edit checking, and customized security parameters. Our RAs enter data remotely in real time to the central database from any of the practice sites. RAVE has extensive built-in reporting capabilities, and data can be exported to standard formats for data analysis (for example, to SAS (Statistical Analysis System) software).. Laboratory tests are directly uploaded to RAVE through a secure web portal.

### Trial monitoring

A Data Monitoring Committee was not deemed necessary, as the experimental intervention (oral iron treatment with 6 mg elemental iron/kg/day given once daily or in two or three divided doses daily for four months plus dietary counseling) is the standard of care for children in the same age group with IDA, and the side effect profile is well known. In the current study of children with NAID, similar side effects are expected and are collected.

### Adverse event reporting

All adverse events will be reported to the Hospital for Sick Children Research Ethics Board, according to the Hospital for Sick Children’s adverse event reporting requirements. All adverse drug reactions to the study medication will be reported to Health Canada within 15 calendar days or, for death or life-threatening events, within seven calendar days. In the latter case, a follow-up report must be filed within eight calendar days. Adverse reactions will be managed according to the Hospital for Sick Children’s standard clinical management practices.

### Statistical analysis

From the screening cohort, the baseline characteristics of the three groups (NAID, IDA, and IS) will be compared with descriptive statistics and significance testing. Categorical variables will be compared with a chi-square test, and continuous variables will be compared with an analysis of variance (ANOVA), or non-parametric equivalent. For participants with NAID randomized to treatment or placebo groups, no significance testing will be performed on the baseline characteristics; however, we will note any imbalances that have arisen by chance which may be clinically meaningful. All children with NAID randomized to treatment or placebo will be analyzed in the group to which they were randomized, following the intention-to-treat principle. In the primary analysis, the difference in developmental and hematologic measures in children with NAID randomized to treatment versus placebo will be assessed using linear regression, with the initial baseline measures included as the adjusting variable (analysis of covariance (ANCOVA) method) [56]. In a secondary analysis, additional covariates of clinical or statistical significance (including parent education and family income) will be included in the model. The primary analysis will be a fixed effects model, ignoring stratification by clinic site. A secondary analysis will include a confirmatory analysis using a mixed effects model, with interventions and MSEL scores as the fixed effect and clinic site as the random effect. The non-randomized groups (IS and IDA) will be compared with the NAID groups for difference in their follow-up developmental and hematological outcome using ANOVA. Although efforts to ensure complete data collection and participant follow-up will be maximized, analytic strategies to handle missing data will include imputation techniques, if appropriate. If more than 20% of participants are lost to follow-up, a per protocol analysis will be carried out in addition to the intention-to-treat analysis.

### Power calculation

#### Clinically meaningful difference in tests of cognition

The minimal clinically important difference (MCID) for the primary outcome of interest (child development as measured by MSEL) has been carefully considered by our research team [57]. In a landmark longitudinal study of infants with IDA compared with IS infants followed from infancy (12 to 23 months) through to 19 years of age, infants with IDA were found to have an eight to nine point cognitive disadvantage in infancy. In a subset of low-income infants the gap widened from 10 points in infancy to 25 points by 19 years [58-61]. Another pivotal study by Walter *et al*. has suggested that the MCID for cognitive difference in children may be as low as six points [62]. Studies in older children have shown that a 15 point cognitive disadvantage at age 11 years conferred a relative risk of 0.79 of being alive 65 years later, and a 30 point disadvantage reduced this to 0.63 [63]. From these studies (and a larger body of literature identifying the association between cognition and education, employment, and health) 15 points or greater is clearly clinically meaningful. However, it is important for a trial to have the power to identify the minimally important difference, which might be as low as a six to eight point difference.

Sample size calculation for the randomized part of the OptEC trial (see Figure 1: schematic of study plan) is based primarily on a presumptive effect estimate of the ELC score, which we considered as an MCID. ELC being a summarized indicator of child cognition, we arrived at a sample size estimate through a sensitivity analysis considering an array of possible MCIDs (six to eight point difference) for children’s cognitive development.

From previous research, it is anticipated that the mean ECL score for children with NAID is 90, and the standard deviation is ± 15 [22,23,64]. To detect a six to eight point difference in post-treatment ELC score, with a power of 80% and a significance level of 5%, a total sample size ranging from 112 to 198 (56 to 99 per group) is required. We targeted an approximate sample of 150 (75 per group). Sample size calculation was performed using the t-test formula [56]. With an estimated prevalence of NAID of 10%, it is anticipated that screening approximately 1,500 children will identify 150 children with NAID to be randomized over a four-year period [2,52,65]. Expecting potential drop-outs and withdrawals to be between 0 and 20%, a total of 180 NAID children will be randomized.

For the non-randomized children (IDA and IS), it is anticipated that the mean ELC score for children with IDA is 85, and the mean developmental score for IS children is 100, and both have an ELC standard deviation of ± 15 [22,23,64]. From the screening cohort of 1,500, it is anticipated that 1 to 2% will have IDA (n = 25), and an equal number of randomly selected children with IS (n = 25) will be sampled for comparison [52,65]. To randomly select children with IS, once a child with IDA is identified, the immediate next child identified with IS who agrees to participate is enrolled in the trial.

### Ethical conduct of the OptEC trial

The OptEC trial was granted ethics approval by The Hospital for Sick Children Research Ethics Board ((REB) file number: 1000027782) on 10 May 2012 and approval is renewed annually by the REB. This study has been registered as a clinical trial (Clinicaltrials.gov identifier: NCT01481766). Written informed consent is obtained from parents of all child participants prior to any data collection. The OptEC trial has different consent forms for the three groups of children (NAID, IDA, and IS) based on their iron status and provision of intervention (see Additional file 4: 4a, 4b and 4c: Consent forms). All data collection forms and supporting documents (iron intake guideline, participant information sheet, and telephone script) were approved by the Hospital for Sick Children REB. Blood results are provided to the child’s physician within 24 to 48 hours. Detailed reports of the MSEL are available in approximately four weeks. Parents and physicians may perceive the opportunity for a cognitive assessment and laboratory testing to be a direct benefit of participation in this study. The investigators considered the inclusion of dietary advice and assessment of outcomes at four months in all randomized children to be consistent with good clinical practice. If at four months children in either the intervention or control group have persistent NAID or have progressed to IDA, they are treated and monitored accordingly by their primary physician.

## Discussion

### An internal pilot study to guide the protocol of the OptEC trial

The estimates used for the calculation of the sample size for the OptEC trial were derived from previous trials which differed from the trial currently being designed; for example, different patient population, small numbers of centers, and different treatment duration [66,67]. Application of prior estimates for power calculation of the current trial may lead to an unnecessarily large trial, or the trial may not be large enough to have sufficient power for detection of a clinically relevant treatment effect [66,68]. Therefore, an internal pilot study was initiated.

### Internal pilot study

An internal pilot is incorporated into the main study design of a randomized controlled trial to obtain important parameter estimates. It forms an integral part of the trial itself and is not a separate study. The protocol of the randomized controlled trial designates the first phase of the trial as a ‘pilot’ phase. These estimated parameters are then used to recalculate the sample size or improve the design and conduct of the clinical trial [66,67]. At the end of the trial, data analyses incorporates those collected during the internal pilot, as well as those collected subsequently. Building an internal pilot study into a clinical trial has very small adverse effect on the significance level [66,69]. Other possible uses of internal pilot data include checking the assumptions regarding adherence of the participants to the study intervention [66,70]. Non-adherence may decrease the probability of detecting treatment differences and affect the interpretation of observed differences. Poor adherence can also jeopardize the outcome of clinical trials by reducing their power. Using data from an internal pilot can provide the anticipated adherence level of participants of a larger clinical trial and can be used to implement strategies to enhance compliance [71,72].

### Aim of the OptEC trial internal pilot study

The objectives of the internal pilot study are: to obtain a reliable estimate of the standard deviation (S_2_) of the primary outcome of the OptEC trial; to obtain the correlation between the baseline and follow-up measurement of the primary outcome; to recalculate the sample size of the OptEC trial using the estimates generated from the internal pilot; and to assess the adherence rate and causes of non-compliance in children enrolled in the pilot study.

### Sample size for the internal pilot study

Several authors have considered approaches to pre-selecting the sample size for internal pilot studies. One approach has shown through simulation that to receive a reliable estimate of the true population parameter, the minimum size for an internal pilot should be at least 10 subjects per treatment group for a two-group randomized study [66]. The sample size for the OptEC trial is approximately 150 (75 per group) NAID subjects to be randomized over a period of four years. Thus, we planned an internal pilot using the first 30 NAID (15 per group) subjects randomized to the two treatments groups.

### Methods

When the trial has assessed the endpoints for the 30 participants in the internal pilot, we will calculate the observed standard deviation within each group and pool them to obtain an estimated standard deviation (S_2_). If S_2_ ≤ 15, the trial will continue as planned, so that the total sample size remains between 112 and 198. However, if S_2_ > 15, we will recalculate the sample size using the new estimate of the standard deviation (S_2_) [66]. Recalculation of the sample will be performed using the ANCOVA method. This method uses the correlation between the baseline and follow-up measurement of the primary outcome in sample size calculation [56].

For assessment of adherence, an adherence rate will be calculated using data from the internal pilot study [72,73]. The four-month follow-up form of the OptEC trial collects data on causes of non-adherence, adverse effects, and an approximate per week rate of missed doses of the study drug. This self-reported measure of missed doses will be used to assess the rate of compliance using a method proposed by Klerk *et al*. [74]. In this method, summaries such as the total number of days in which no doses were taken, the length of the monitored interval, and the overall percentage of prescribed doses taken is used to calculate a rate of adherence [73,74]. Adherence rate will be described by placing participants into broad bands, with the percentage of patients in each band. The causes of non-adherence will be identified and summarized as percentage.

### Knowledge translation

Findings from this research will be disseminated directly to the physician participants and to their patients. An annual meeting of all the TARGet Kids! Practice staff (physicians, nurses, and office staff), research team (investigators, research assistants, and students), and policy leaders (representatives from Section of Community Pediatrics, Department of Family and Community Medicine, and parent representatives) will occur. Parents of participants will receive the summary of their child’s developmental assessment, anthropometric measures, and laboratory measures, leading to a direct benefit for individual participants. Further downstream dissemination to primary care physicians will occur through formal and informal venues at local levels, such as educational rounds (for example City Wide Pediatric Rounds and SickKids Annual Pediatric Update) and held by local physician groups. End of grant knowledge will be shared with the academic community through publication in relevant journals and presentations at national and international conferences (Annual Meetings of the Pediatric Academic Societies, and the Canadian Paediatric Society), and locally through hospital rounds and presentations, and through our TARGet Kids! website [75]. Messages will be relevant to professionals working in the fields of pediatrics, family medicine, developmental pediatrics, nutrition, nursing, dietetics, and public health. We will also share our findings with colleagues at the Canadian Paediatric Society and the American Academy of Pediatrics. The principal applicant is a member of the Canadian Task Force for Preventive Health Care and will participate in the upcoming guideline development for developmental screening and screening for IDA. Opportunities for coverage in lay publications and media will be sought using an experienced knowledge broker at SickKids Department of Public Relations.

### Trial status

The trial has been recruiting since July 2012. The end of recruitment is estimated to be June 2016.

## Additional files

Additional file 1:
**The SPIRIT checklist.**
Additional file 2:
**Iron intake guideline.**
Additional file 3:
**4 month follow-up form.**
Additional file 4:
**4a, 4b and 4c: Consent forms.**

## Abbreviations

CBQ: Children’s Behavior Questionnaire
CRP: C-reactive protein
ECBQ: Early Childhood Behavior Questionnaire
ELC: Early Learning Composite
IBQ: Infant Behavior Questionnaire
IDA: Iron deficiency anemia
IS: Iron sufficiency
MSEL: Mullen Scale of Early Learning
MSS: Mount Sinai Services
NAID: Non-anemic iron deficiency
RA: Research assistant.
