# Supplementary material for: Optimizing early child development for young children with non-anemic iron deficiency in the primary care practice setting (OptEC): study protocol for a randomized controlled trial
Source: Trials. 2015 Apr 2;16:132. doi: 10.1186/s13063-015-0635-z (PMC4389731; doi:10.1186/s13063-015-0635-z)
Supplement: Additional file 1: — The SPIRIT checklist. [file 13063_2015_635_MOESM1_ESM.pdf]

Food Guide for Pre-school Children to Improve Iron In-take

**Types of food rich in iron (According to food types containing high to low level of iron)**	**Food types**	**Number of servings/day**	**Examples**
	Meat and eggs<br>(highest source of iron)	**1 serving**<br>1 serving =<br>• 1/2 cup cooked fish/poultry/lean meat<br>**OR**<br>• 2 eggs	• Beef (burger, beef liver, corned beef, steak)<br>• Chicken and turkey (breast, thigh, wings, liver)<br>• Fish (haddock, halibut, salmon, tuna) fresh or canned in water<br>• Lamb and pork<br>• Eggs (especially egg yolks)
	Meat alternatives<br>(medium source of iron)	**1 serving**<br>1 serving = 3/4 cup cooked beans or tofu	• Beans (chick peas, lima beans, navy beans, kidney beans, lentils); Baked beans (canned)<br>• Tofu (firm)
	Grain Products<br>(lower source of iron)	**3 servings**<br>1 serving =<br>• 3/4 cup of cereal/oat–meal / cream of wheat<br>**OR**<br>• 1/2 cup cooked pasta/ rice	• Cream of wheat; Oatmeal; Iron-enriched breakfast cereals (cheerios, corn flakes)<br>• Enriched pasta<br>• Enriched Rice
	Vegetables and Fruit<br>(lower source of iron)	**4 servings**<br>1 serving =<br>• 1/2 cup of fresh, frozen or canned vegetable/fruit/ fruit juice<br>**OR**<br>• 1 cup of leafy raw vegetable	• Broccoli<br>• Spinach<br>• Baked potato with skin<br>• Fresh or dried fruits (apricots, figs, raisins)<br>• Prune juice

**Foods that increase iron absorption**	Along with foods rich in iron **4 servings/day** of vegetable and fruits that contain vitamin C is essential<br><br>1 serving =<br>• 1/2 cup of fresh, frozen or canned vegetable/fruit/ fruit juice<br><br>**OR**<br><br>• 1 cup of leafy raw vegetable		**Vitamin C containing foods:**<br>• Citrus fruits (orange, grapefruit, tomatoes) and juices<br>• Cantaloupe<br>• Kiwifruit<br>• Leafy greens (spinach, cabbage), cauliflower<br>• Broccoli, Brussels sprouts<br>• Green and red peppers
**Dietary habits that prevent development of iron deficiency**			• Limit cow's milk to 2 cups (16 ounces, or 450 mL) per day<br>• Limit juice to 1/2 to 1 cup (4 to 8 ounces, or 115 to 225 mL) per day<br>• Remove all baby bottles and offer milk, juice and water from a cup<br>• Do not give any tea. Commercial black tea contains substances that bind to iron so it cannot be used by the body.
**Tips to increase iron intake**			• Adding beef to tomato or pasta sauce<br>• Adding chunks of ham to macaroni and cheese<br>• Serving baked beans with pork and tomato sauce<br>• Sprinkling dried fruit (dates, raisins, prunes, apricots) on cereal<br>• Add dried peas or beans to soups and casseroles

References:

- Health information for families from The Hospital for Sick Children. URL: www.aboutkidshealth.ca (accessed on September 26, 2011).
- Eating well with Canada's Food Guide. URL: www.hc-sc.gc.ca/fn-an/alt_formats/hpfb-dgpsa/pdf/food-guide-aliment/view_eatwell_vue_bienmang-eng.pdf (accessed on September 26, 2011).
- Loading Up with Iron-rich Foods. Nutrition Matters. Toronto Public Health. URL: www.toronto.ca/health (accessed on September 26, 2011).

- Nutrition for healthy term infants: Recommendations from 6 to 24 months. Health Canada, Canadian Paediatric Society, Dietitians of Canada and Breastfeeding Committee of Canada; 2013-2014 [cited 2014 August 19th]; Available from: http://www.hc-sc.gc.ca/fn-an/nutrition/infant-nourisson/recom/recom-6-24-months-6-24-mois-eng.php.
