# Supplementary material for: Optimizing early child development for young children with non-anemic iron deficiency in the primary care practice setting (OptEC): study protocol for a randomized controlled trial
Source: Trials. 2015 Apr 2;16:132. doi: 10.1186/s13063-015-0635-z (PMC4389731; doi:10.1186/s13063-015-0635-z)
Supplement: Additional file 2: — Iron intake guideline. [file 13063_2015_635_MOESM2_ESM.pdf]

OptEC Trial: Optimizing early child development in the primary care practice setting: Pragmatic randomized trial of iron treatment for young children with non-anemic iron deficiency

Follow-up Data Collection Form – Data linking sheet
(To be stored separately from study data)

ID String # ________________ Date ________________________

Age _____ (months) Child's Sex: Male [ ] Female [ ]

Home Telephone #___________________ Work/cell Tel # _____________________

Name of caregiver interviewed ____

Relationship to child ____

Office use only
ID_______________

OptEC Trial: Optimizing early child development in the primary care practice setting: Pragmatic randomized trial of iron treatment for young children with non-anemic iron deficiency (NAID)

Follow-up Data Collection Form

1. How many days last week did your child receive the provided study drug **(circle one)**?

   0 1 2 3 4 5 6 7

2. Since starting this study, has your child taken any iron supplements regularly other than the ones provided for this study **(fill in all that apply)**?

- No
- Iron: Ferinsol _______ ml per ____________ (day, week, month, year)
- Iron: Other _______ ml per ____________ (day, week, month, year)
- Multivitamin with iron _______ ml per ____________ (day, week, month, year)

3. Since starting this study, has your child taken any other vitamins or supplements regularly **(fill in all that apply)?**

- No
- Vitamin D: Drops _______ ml per ____________ (day, week, month, year)
- Vitamin D: Liquid _______ ml per ____________ (day, week, month, year)
- Multivitamin (without iron) _______ ml per ____________ (day, week, month, year)
- Other—**Please explain**____

4. How hard or easy has it been to give the provided study drug?

   Easy ... Hard

   0 1 2 3 4 5

5. Did your child like taking the study drug **(circle one)**?

- Yes
- No
- Indifferent

6. In a typical week, how many **<u>days</u>** did your child receive the study drug **(please circle)**?

   0 1 2 3 4 5 6 7

7. If your child received the study drug 6days/week or less, please state the reason **(circle all that apply)**?

• Takes too long	Yes	No
• Too messy	Yes	No
• My child didn’t like it	Yes	No

• Not convinced that it will benefit my child	Yes	No
• Too hard to give it	Yes	No
• Forgot	Yes	No
• Other reason(s)	Yes	No

8. Did your child experience any of the following while administering the study drug **(circle all that apply)**?

• Coughing	Yes	No
• Spitting up	Yes	No
• Choking, gagging	Yes	No
• Unhappy with the taste	Yes	No

9. Did your child experience any of the following during the past 4 months (**circle all that apply)**?

• Staining of the teeth	Yes	No
• Constipation	Yes	No
• Loose stool	Yes	No
• Passage of black stool	Yes	No

10. Is your child **currently** breastfeeding **(please circle)**?

- Yes
- No — at what age did you stop breastfeeding? ________________ months
- Not applicable, did not breastfeed

11. Please specify your child's diet for the past 3 days. **Please check all that apply.**

- ☐ Breast milk
- ☐ Infant formula
- ☐ Red meat (beef, veal, pork, lamb, etc.)
- ☐ Poultry (chicken, turkey, duck, etc.)
- ☐ Fish (salmon, halibut, haddock, cod, tuna, etc.)
- ☐ Shellfish (lobster, crab, shrimp, etc.)
- ☐ Eggs
- ☐ Milk ☐ **Skim** ☐ **1%** ☐ **2%** ☐ **Homo**
- ☐ Fruits
- ☐ Vegetables
- ☐ Cheese
- ☐ Yogurt
- ☐ Margarine
- ☐ Honey
- ☐ Whole grain products (bread, bagel, bun, cereal, pasta, rice, roti, tortillas, etc.)
- ☐ Fast Food
- ☐ Infant cereal
- ☐ Vegetarian: does not eat red meat, poultry, fish or shellfish
- ☐ Vegan: does not eat red meat, poultry, fish, shellfish, eggs, dairy or honey

12. Circle how many cups of each drink your child has <u>currently in a typical day.</u> (1 cup = 8 ounces= 250 ml)

Cow's milk			0	½	1	2	3	4	5+
Infant formula			0	½	1	2	3	4	5+
Infant cereal	0	¼	½	¾	1	2	3	4	5+
Soy milk			0	½	1	2	3	4	5+
Other milk (rice, goat etc)			0	½	1	2	3	4	5+
100% Juice (apple, orange etc)			0	½	1	2	3	4	5+
Sweetened drinks (Kool aid, Sunny D, etc.)			0	½	1	2	3	4	5+
Tea			0	½	1	2	3	4	5+
Soda or Pop			0	½	1	2	3	4	5+

13. Did your child's diet include the following foods during the last 4 months? **Please check all that apply**?

☐ Whole grain products (example – iron enriched breakfast cereals, enriched pasta and rice, beans such as chick peas, kidney beans, lentils and canned baked beans)

_______ times per ____________ (day, week, month)

☐ Tofu _______ times per ____________ (day, week, month)

☐ Citrus fruits (example - oranges, grapefruit, lemon juice, tomatoes, cantaloupe, kiwi fruit)

_______ times per ____________ (day, week, month)

☐ Citrus vegetables (example – spinach, cabbage, broccoli, Brussels sprouts, bell pepper, cauliflower)

_______ times per ____________ (day, week, month)

14. Has your child been ill within the **past 4 months**?

☐ No

☐ Yes, (complete all that apply below)

- Colds or flus, how many times? ____
- Asthma attack, how many times? ____
- Pneumonia, how many times? ____
- Ear infection, how many times? ____

For office use only

Height ___________ cm

Weight ___________ kg

BMI ___________ kg/m^2

Waist circumference ________ cm
