# Supplementary material for: Optimizing early child development for young children with non-anemic iron deficiency in the primary care practice setting (OptEC): study protocol for a randomized controlled trial
Source: Trials. 2015 Apr 2;16:132. doi: 10.1186/s13063-015-0635-z (PMC4389731; doi:10.1186/s13063-015-0635-z)
Supplement: Additional file 4: — 4a, 4b and 4c: Consent forms. [file 13063_2015_635_MOESM4_ESM.zip › Additional file 4/Consent forms/Consent form_IDA.doc]

# RESEARCH CONSENT FORM

# (For Parents of children with Iron Deficiency Anemia)

**Study Title:** OptEC: Optimizing early child development in the primary care practice setting: Pragmatic randomized trial of iron treatment for young children with non-anemic iron deficiency (NAID)

**Principal/Qualified Investigator:**

Dr. Patricia Parkin	Ph: (416) 813-6933

**Sub/Co-investigators**:

Dr. Catherine Birken	Ph: (416)813-6933	Dr. Darcy Fehling	Ph: (416)424-3822

Dr. Jonathon Maguire	Ph: (416)919-3462	Dr. Muhammad Mamdani	Ph:(416)864-3037

Dr Eva Mamak	Ph: (416)813-6784	Dr. Colin Macarthur	Ph:(416)813-7654 Ex.28474

Kevin Thorpe	Ph: (416)864-5776	Dr. Stanley Zlotkin	Ph:(416) 813-8056

Dr. Kawsari Abdullah	Ph: (416) 813-8144

**Clinical Research Coordinators:**

Julie DeGroot	Ph: (416) 813-8144

Dr. Marina Khovratovich	Ph: (416) 813-2129

**Purpose of the Research:**

Your child’s doctor is a member of TARGet Kids! (Toronto Area Research Group) which is a group of child health researchers from SickKids and St. Michael’s Hospitals and community doctors dedicated to improving the health of young children.

Previous research has shown that children with later stages of iron deficiency (low blood iron levels) called iron deficiency anemia (IDA) may have serious delays in their development and early learning. About 1-2% of healthy Toronto children have this condition. However, not very much is known about the effects of early stages of iron deficiency (called non-anemic iron deficiency or “NAID”) which we have found in about 10% of healthy Toronto children. Currently doctors do not routinely test for iron deficiency in young children. We don’t know if screening children will result in better outcomes but we are trying to find this out. It is common practice for parents of pre-school children at risk for early stage iron deficiency to receive diet counselling to improve the iron intake from the child’s diet. However, it is not known whether it is necessary to give children an oral iron supplement. We are doing this research study to find out if children require oral iron or if diet counseling alone is effective to treat iron deficiency. We also like to compare the health and development of children identified with late and early stages of iron deficiency and also with children having normal iron level.

**Description of the Research:**

You are being invited to participate in this research study because your child was found to have late stage of iron deficiency anemia (IDA) on the blood tests as part of her/his participation in TARGet Kids!. Thus it is very important that your child receives oral iron in order to increase his/her iron level. If you agree to participate in our study, your child will receive oral iron drop and diet counseling to improve iron intake from your child’s diet which is our current standard of practice.

Your child should take the iron drop by mouth twice a day for 4 months. Iron dosages in this study are safe and within the level recommended by Health Canada for children. Your child’s doctor will instruct you on how much drop you need to give to your child every day. During the study we ask that your child doesn’t take any iron supplements or multivitamins that contain iron until your child has come for their follow-up visit after 4 months.

All parents and children will receive a list of foods that are a good source of iron. You will also receive advice on the amount of cow’s milk your child should get each day and which foods to avoid because they lower the amount of iron the body absorbs.

If you agree to participate you will be asked to return to the physician’s office. During this visit your child will be tested for his/her developmental status. This will be performed by a trained psychometrist. It will take approximately 45-60 minutes. Developmental testing does not require any invasive procedure. Your child will do activities like playing with blocks, looking at pictures, and playing with different toys and the psychometrist will observe your child to determine the activities they are capable of at this time. You will remain with your child through the entire procedure. An appointment for your child’s follow-up visit at four months will be scheduled by a research assistant on the day of your child’s first developmental testing.

After 4 months you (preferably the same parent from the first visit) will be asked to return to your doctor’s office for your child’s follow-up visit. We will ask you to return your bottle. During this visit your child will again have blood taken to find out his/her iron level and undergo developmental testing. About 3-5 mL or 1 teaspoon of blood will be collected by a trained health professional experienced in taking blood from children. The blood sample will be sent to Mount Sinai Hospital, Toronto, for testing but no one will know your child’s identity. The blood test and the developmental testing may occur on the same day or you can continue your follow-up visit at a later scheduled time.

At your follow-up visit you will also be asked to complete a follow-up questionnaire. This questionnaire will contain questions regarding your experience and your child’s responses to taking the iron drop. It will also include questions regarding the dietary intake of your child just prior to your follow-up visit to the doctor’s office. We will also look at your child’s health records. Your child will be in this study for a total of 4 months.

We will include 25 children who have iron deficiency anemia in this study. We will compare these children with 25 children who have normal iron levels who do not need treatment. We will also compare these two groups with about 150 children from several doctors’ offices who will receive diet counseling and iron drop or placebo (drop with no active medicine). In total, about 200 children will take part in this study.

**Potential Harms:**

Taking iron drop by mouth may sometimes cause side effects that include the staining of teeth, constipation and passing black stools. If your child takes the iron as directed by the study doctor he/she may experience all of these side effects or some of them during the four months and for a few days after stopping the iron drop. However, these symptoms are completely reversible and non-harmful and you are encouraged to continue the treatment if these symptoms develop. Also the study drug we provide may have a metallic taste which your child may not like. Therefore we will provide you with an “information sheet” that will contain instructions on how you should administer the study drug to your child. These are the known effects of taking iron, but there may be harms we don’t know about.

There may be a small amount of bleeding when blood is taken from a vein and there may be slight discomfort, bruising or redness that will usually disappear in a few days. Blood collection is usually a quick process (about 5 minutes) and at other times it can require a little more time. Your child can have some numbing cream put on their skin before the blood test.

**Potential Discomforts or Inconveniences:**

You and your child will need to return to your doctor’s office for 2 extra visits. The first visit will be for the developmental tests and this will take 45 to 60 minutes. The second visit will be 4 months later to repeat blood tests and developmental testing. Again this second visit will take about 60 minutes.

**Potential Benefits:**

**To individual subjects:**

Your child may benefit from participating in this study by having his or her blood iron level measured and by you knowing the developmental status of your child. Results of these tests will be provided to your child’s doctor with your permission. This could inform your doctor if your child might need an ongoing iron supplement or further developmental testing or interventions. The results of the trial will be made available to you through the TARGet Kids! Newsletter, which can be available at your physician’s office. You may also contact the Principal Investigator if you wish to obtain more detailed study results.

**To society:**

The major benefit of this study will be to understand whether screening and treating young children for iron deficiency can improve their later development. This information would be very important to parents, children and health care providers across the country and internationally. The information we learn may help improve the health of children across Canada.

**Alternatives to Participation:**

If you choose for your child to not take part in this study, your child’s doctor will decide what treatment is best for your child. This may or may not include an iron supplement.

**Confidentiality:**

We will respect your privacy. No information about who your child is will be given to anyone or be published without your permission, unless required by law. For example, the law could make us give information about you if a child has been abused, if your child has an illness that could spread to others, if your child or someone else talks about suicide (killing themselves or others), or if the court orders us to give them the study papers.

SickKids Clinical Research Monitors, employees of the funder (CIHR) or sponsor of the study, or the regulator of the study may see your child’s health records to check on the study. For example, people from Health Canada Health Products and Food Branch, if necessary, may look at your child’s records.

By signing this consent form, you agree to let these people look at your child’s records. We will put a copy of this research consent form in your child’s patient health records. We will give you a copy for your files.

Your child’s blood samples will be sent to Mount Sinai Hospital, Toronto, for testing. No one will know the identity of your child.

The data produced from this study will be stored in a secure, locked location. Only members of the research team (and maybe those described above) will have access to the data. This could include external research team members. Following completion of the research study, the data will be kept as long as required then destroyed as required by SickKids policy. Published study results will not reveal your child’s identity.

The results of the tests we describe in this form will be used only for this study. If another health care professional caring for your child needs to see these results, you will have to give us your permission. We will ask you to sign a form saying that you agree that this person can see your child’s results. We recommend that only a registered psychologist or doctor tell you what the results of these tests mean.

**Reimbursement:**

We will reimburse you for all your reasonable out of pocket expenses for being in this study. If you stop taking part in the study, we will pay you for your expenses for taking part in the study up until that point.

**Participation:**

It is your choice to allow your child to take part in this study. You can stop at any time. The care your child receives at SickKids, St. Michael’s Hospital and at your child’s doctor’s office will not be affected in any way by whether you take part in this study.

New information that we get while we are doing this study may affect your decision for your child to take part in this study. If this happens, we will tell you about this new information. And we will ask you again if you still want your child to be in the study.

During this study we may create new tests, new medicines, or other things that may be worth some money. Although we may make money from these findings, we cannot give your child any of this money now or in the future because your child took part in this study.

In some situations, the study doctor or the people paying for the study may decide to stop the study. This could happen even if the drug given in this study is helping your child. If this happens, the study doctor will talk to you about what will happen next.

If your child becomes ill or is harmed because of study participation, we will treat your child for free. Your signing this consent form does not interfere with your legal rights in any way. The staff of the study, any people who gave money for the study, the hospitals, and the doctors’ offices are still responsible, legally and professionally, for what they do.

**Sponsorship:**

Funding for this TARGet Kids study is provided by the Canadian Institute of Health Research. The sponsor of this study is Dr. Patricia Parkin and The Hospital for Sick Children.

**Conflict of Interest:**

Dr. Parkin and the other research team members have no conflict of interest to declare.

**Future Research**:

In the future, our research team may approach you to participate in other studies with the aim of improving children’s health. The research will be explained to you and your consent will be asked again at that time.

**Consent**:

By signing this form, I agree that:

1) You have explained this study to me. You have answered all my questions.

2) You have explained the possible harms and benefits (if any) of this study.

3) I know what I could do instead of having my child take part in this study. I understand that I

have the right to refuse to let my child take part in the study. I also have the right to take my

child out of the study at any time. My decision about my child taking part in the study will not

affect my child’s health care at Sick Kids, St. Michael’s or my child’s pediatrician or family

doctor office.

4) I am free now, and in the future, to ask questions about the study.

5) I have been told that my child’s medical records will be kept private except as described to me.

6) I understand that no information about my child will be given to anyone or be published without

first asking my permission.

7) I have read and understood pages 1 to 5 of this consent form. I agree, or consent, that my

child _________________________________________ may take part in this study.

__________________________________	_______________________________	_______

Printed Name of Parent/Legal Guardian	Parent/Legal Guardian’s signature	date

_______________________________________	___________________________________	________

Printed Name of person who explained consent	Signature of Person who explained consent	date

_______________________________________	___________________________________	________

Printed Witness’ name (if the parent/legal guardian	Witness’ signature	date

does not read English)

If you have any questions about the TARGet Kids study, please call Dr Patricia Parkin at (416) 813-6933

If you have questions about your child’s rights as a subject in a study or injuries during a study, please call the Research Ethics Manager at 416-813-5718.
